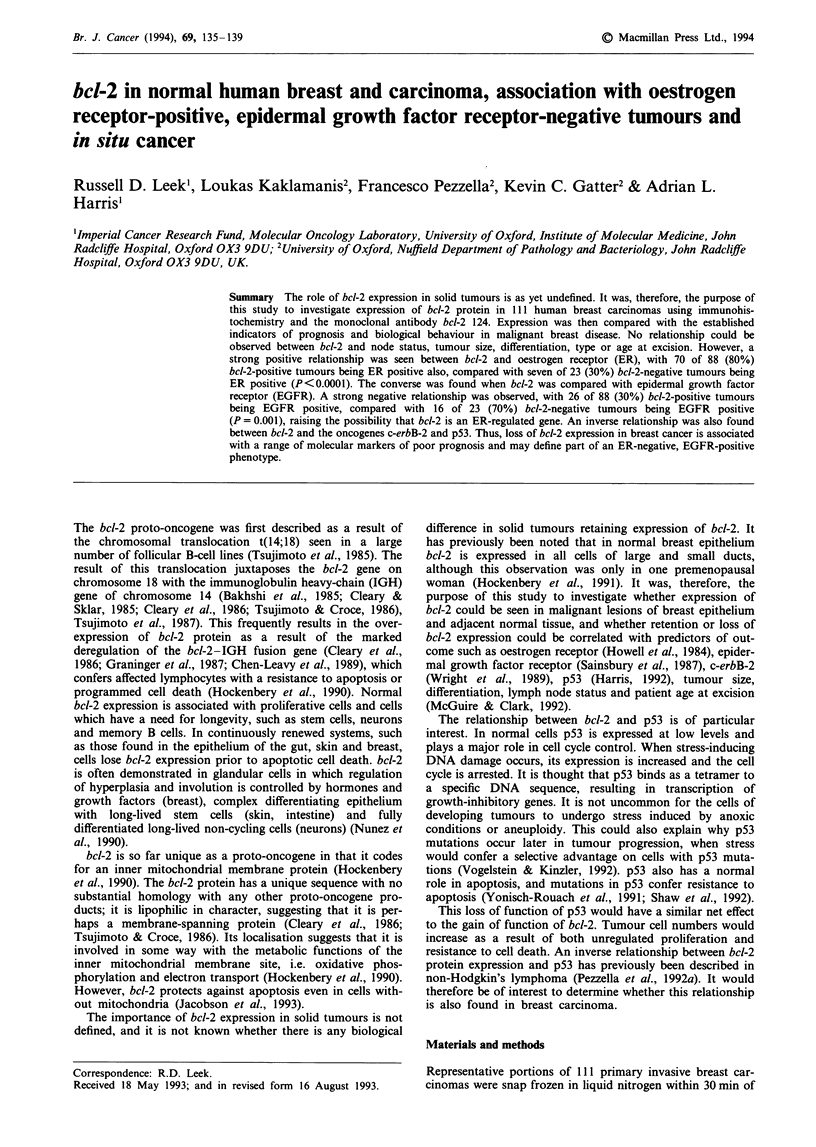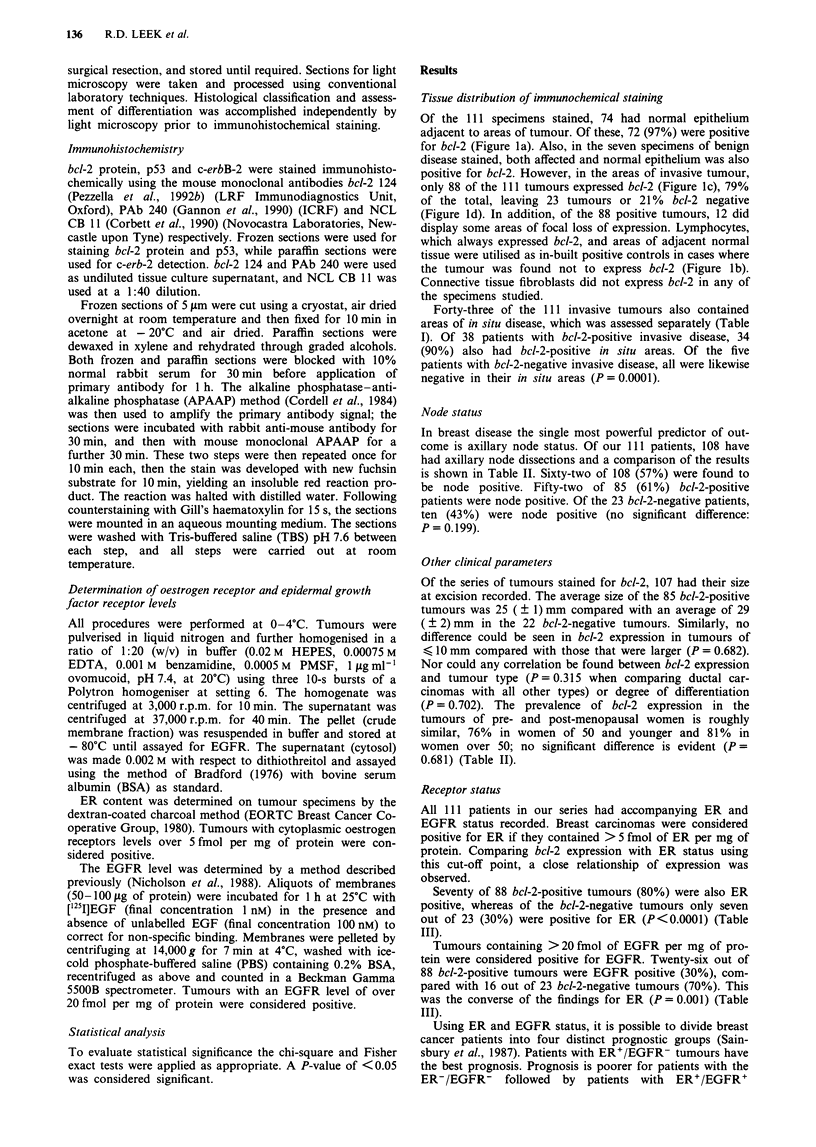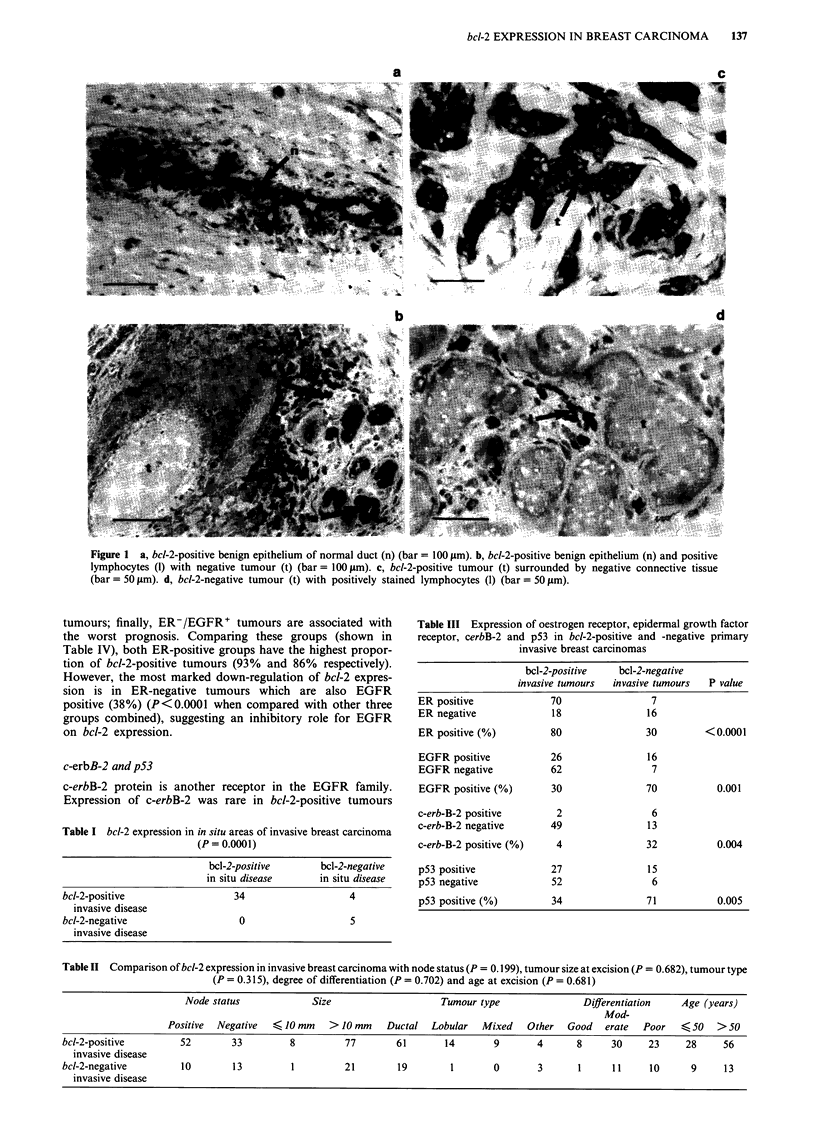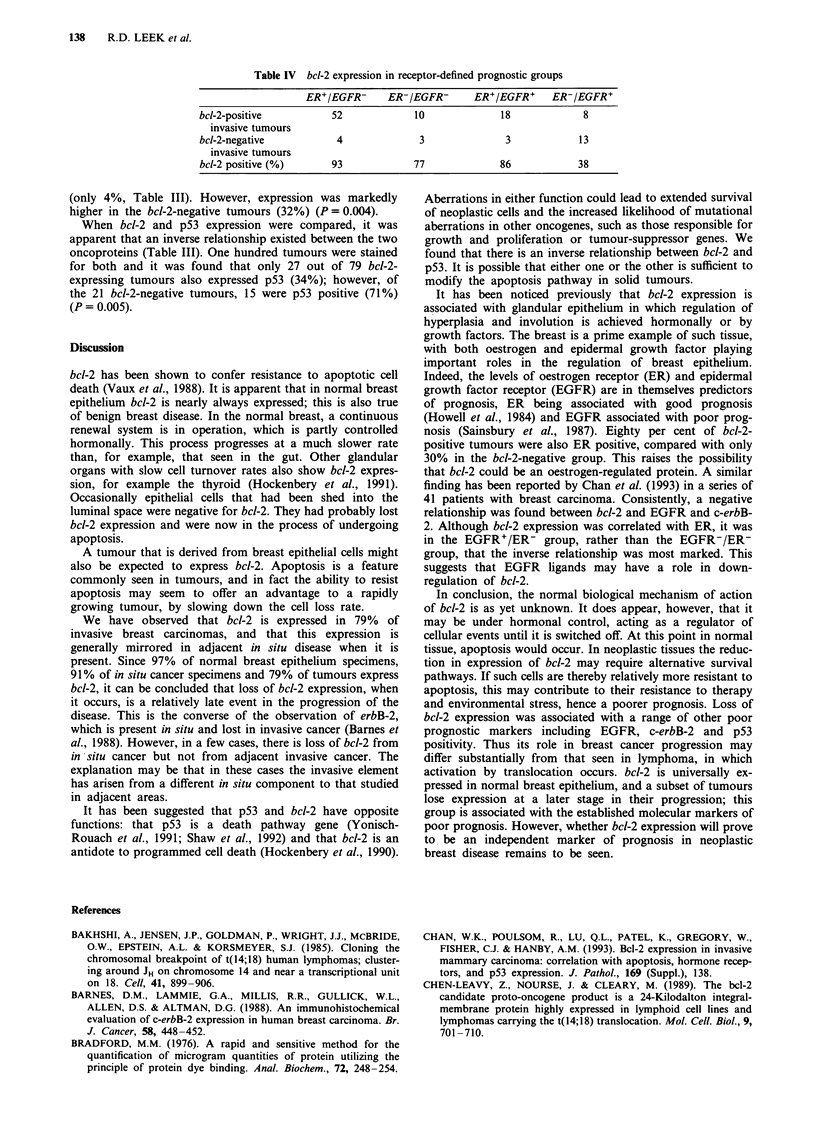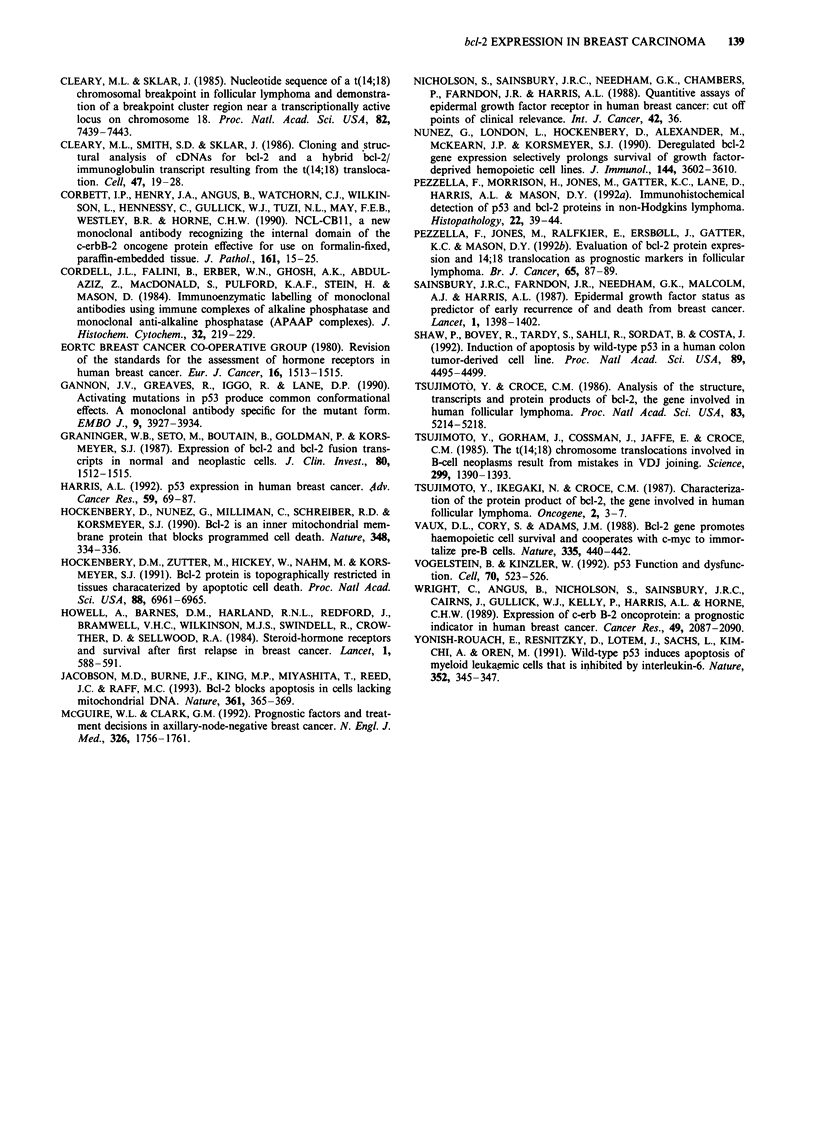# bcl-2 in normal human breast and carcinoma, association with oestrogen receptor-positive, epidermal growth factor receptor-negative tumours and in situ cancer.

**DOI:** 10.1038/bjc.1994.22

**Published:** 1994-01

**Authors:** R. D. Leek, L. Kaklamanis, F. Pezzella, K. C. Gatter, A. L. Harris

**Affiliations:** Imperial Cancer Research Fund, University of Oxford, John Radcliffe Hospital, UK.

## Abstract

**Images:**


Br. J. Cancer (1994), 69, 135-139 © Macmillan Press Ltd., 1994

### *bcl*-2 in normal human breast and carcinoma, association with oestrogen receptor-positive, epidermal growth factor receptor-negative tumours and *in situ* cancer

Russell D. Leek[1], Loukas Kaklamanis[2], Francesco Pezzella[2], Kevin C. Gatter[2] & Adrian L. Harris[1]

[1]*Imperial Cancer Research Fund, Molecular Oncology Laboratory, University of Oxford, Institute of Molecular Medicine, John Radcliffe Hospital, Oxford OX3 9DU;* [2]*University of Oxford, Nuffield Department of Pathology and Bacteriology, John Radcliffe Hospital, Oxford OX3 9DU, UK.*

**Summary** The role of *bcl*-2 expression in solid tumours is as yet undefined. It was, therefore, the purpose of this study to investigate expression of *bcl*-2 protein in 111 human breast carcinomas using immunohistochemistry and the monoclonal antibody *bcl*-2 124. Expression was then compared with the established indicators of prognosis and biological behaviour in malignant breast disease. No relationship could be observed between *bcl*-2 and node status, tumour size, differentiation, type or age at excision. However, a strong positive relationship was seen between *bcl*-2 and oestrogen receptor (ER), with 70 of 88 (80%) *bcl*-2-positive tumours being ER positive also, compared with seven of 23 (30%) *bcl*-2-negative tumours being ER positive ($P < 0.0001$). The converse was found when *bcl*-2 was compared with epidermal growth factor receptor (EGFR). A strong negative relationship was observed, with 26 of 88 (30%) *bcl*-2-positive tumours being EGFR positive, compared with 16 of 23 (70%) *bcl*-2-negative tumours being EGFR positive ($P = 0.001$), raising the possibility that *bcl*-2 is an ER-regulated gene. An inverse relationship was also found between *bcl*-2 and the oncogenes c-*erb*B-2 and p53. Thus, loss of *bcl*-2 expression in breast cancer is associated with a range of molecular markers of poor prognosis and may define part of an ER-negative, EGFR-positive phenotype.

The *bcl*-2 proto-oncogene was first described as a result of the chromosomal translocation t(14;18) seen in a large number of follicular B-cell lines (Tsujimoto *et al.*, 1985). The result of this translocation juxtaposes the *bcl*-2 gene on chromosome 18 with the immunoglobulin heavy-chain (IGH) gene of chromosome 14 (Bakhshi *et al.*, 1985; Cleary & Sklar, 1985; Cleary *et al.*, 1986; Tsujimoto & Croce, 1986), Tsujimoto *et al.*, 1987). This frequently results in the over-expression of *bcl*-2 protein as a result of the marked deregulation of the *bcl*-2-IGH fusion gene (Cleary *et al.*, 1986; Graninger *et al.*, 1987; Chen-Leavy *et al.*, 1989), which confers affected lymphocytes with a resistance to apoptosis or programmed cell death (Hockenbery *et al.*, 1990). Normal *bcl*-2 expression is associated with proliferative cells and cells which have a need for longevity, such as stem cells, neurons and memory B cells. In continuously renewed systems, such as those found in the epithelium of the gut, skin and breast, cells lose *bcl*-2 expression prior to apoptotic cell death. *bcl*-2 is often demonstrated in glandular cells in which regulation of hyperplasia and involution is controlled by hormones and growth factors (breast), complex differentiating epithelium with long-lived stem cells (skin, intestine) and fully differentiated long-lived non-cycling cells (neurons) (Nunez *et al.*, 1990).

*bcl*-2 is so far unique as a proto-oncogene in that it codes for an inner mitochondrial membrane protein (Hockenbery *et al.*, 1990). The *bcl*-2 protein has a unique sequence with no substantial homology with any other proto-oncogene products; it is lipophilic in character, suggesting that it is perhaps a membrane-spanning protein (Cleary *et al.*, 1986; Tsujimoto & Croce, 1986). Its localisation suggests that it is involved in some way with the metabolic functions of the inner mitochondrial membrane site, i.e. oxidative phosphorylation and electron transport (Hockenbery *et al.*, 1990). However, *bcl*-2 protects against apoptosis even in cells without mitochondria (Jacobson *et al.*, 1993).

The importance of *bcl*-2 expression in solid tumours is not defined, and it is not known whether there is any biological difference in solid tumours retaining expression of *bcl*-2. It has previously been noted that in normal breast epithelium *bcl*-2 is expressed in all cells of large and small ducts, although this observation was only in one premenopausal woman (Hockenbery *et al.*, 1991). It was, therefore, the purpose of this study to investigate whether expression of *bcl*-2 could be seen in malignant lesions of breast epithelium and adjacent normal tissue, and whether retention or loss of *bcl*-2 expression could be correlated with predictors of outcome such as oestrogen receptor (Howell *et al.*, 1984), epidermal growth factor receptor (Sainsbury *et al.*, 1987), c-*erb*B-2 (Wright *et al.*, 1989), p53 (Harris, 1992), tumour size, differentiation, lymph node status and patient age at excision (McGuire & Clark, 1992).

The relationship between *bcl*-2 and p53 is of particular interest. In normal cells p53 is expressed at low levels and plays a major role in cell cycle control. When stress-inducing DNA damage occurs, its expression is increased and the cell cycle is arrested. It is thought that p53 binds as a tetramer to a specific DNA sequence, resulting in transcription of growth-inhibitory genes. It is not uncommon for the cells of developing tumours to undergo stress induced by anoxic conditions or aneuploidy. This could also explain why p53 mutations occur later in tumour progression, when stress would confer a selective advantage on cells with p53 mutations (Vogelstein & Kinzler, 1992). p53 also has a normal role in apoptosis, and mutations in p53 confer resistance to apoptosis (Yonisch-Rouach *et al.*, 1991; Shaw *et al.*, 1992).

This loss of function of p53 would have a similar net effect to the gain of function of *bcl*-2. Tumour cell numbers would increase as a result of both unregulated proliferation and resistance to cell death. An inverse relationship between *bcl*-2 protein expression and p53 has previously been described in non-Hodgkin's lymphoma (Pezzella *et al.*, 1992*a*). It would therefore be of interest to determine whether this relationship is also found in breast carcinoma.

### Materials and methods

Representative portions of 111 primary invasive breast carcinomas were snap frozen in liquid nitrogen within 30 min of

Correspondence: R.D. Leek.
Received 18 May 1993; and in revised form 16 August 1993.

surgical resection, and stored until required. Sections for light microscopy were taken and processed using conventional laboratory techniques. Histological classification and assessment of differentiation was accomplished independently by light microscopy prior to immunohistochemical staining.

*Immunohistochemistry*

*bcl*-2 protein, p53 and c-*erb*B-2 were stained immunohistochemically using the mouse monoclonal antibodies *bcl*-2 124 (Pezzella *et al.*, 1992*b*) (LRF Immunodiagnostics Unit, Oxford), PAb 240 (Gannon *et al.*, 1990) (ICRF) and NCL CB 11 (Corbett *et al.*, 1990) (Novocastra Laboratories, Newcastle upon Tyne) respectively. Frozen sections were used for staining *bcl*-2 protein and p53, while paraffin sections were used for c-*erb*-2 detection. *bcl*-2 124 and PAb 240 were used as undiluted tissue culture supernatant, and NCL CB 11 was used at a 1:40 dilution.

Frozen sections of 5 μm were cut using a cryostat, air dried overnight at room temperature and then fixed for 10 min in acetone at − 20°C and air dried. Paraffin sections were dewaxed in xylene and rehydrated through graded alcohols. Both frozen and paraffin sections were blocked with 10% normal rabbit serum for 30 min before application of primary antibody for 1 h. The alkaline phosphatase–anti-alkaline phosphatase (APAAP) method (Cordell *et al.*, 1984) was then used to amplify the primary antibody signal; the sections were incubated with rabbit anti-mouse antibody for 30 min, and then with mouse monoclonal APAAP for a further 30 min. These two steps were then repeated once for 10 min each, then the stain was developed with new fuchsin substrate for 10 min, yielding an insoluble red reaction product. The reaction was halted with distilled water. Following counterstaining with Gill's haematoxylin for 15 s, the sections were mounted in an aqueous mounting medium. The sections were washed with Tris-buffered saline (TBS) pH 7.6 between each step, and all steps were carried out at room temperature.

*Determination of oestrogen receptor and epidermal growth factor receptor levels*

All procedures were performed at 0–4°C. Tumours were pulverised in liquid nitrogen and further homogenised in a ratio of 1:20 (w/v) in buffer (0.02 M HEPES, 0.00075 M EDTA, 0.001 M benzamidine, 0.0005 M PMSF, 1 $\mu g\,ml^{-1}$ ovomucoid, pH 7.4, at 20°C) using three 10-s bursts of a Polytron homogeniser at setting 6. The homogenate was centrifuged at 3,000 r.p.m. for 10 min. The supernatant was centrifuged at 37,000 r.p.m. for 40 min. The pellet (crude membrane fraction) was resuspended in buffer and stored at − 80°C until assayed for EGFR. The supernatant (cytosol) was made 0.002 M with respect to dithiothreitol and assayed using the method of Bradford (1976) with bovine serum albumin (BSA) as standard.

ER content was determined on tumour specimens by the dextran-coated charcoal method (EORTC Breast Cancer Cooperative Group, 1980). Tumours with cytoplasmic oestrogen receptors levels over 5 fmol per mg of protein were considered positive.

The EGFR level was determined by a method described previously (Nicholson *et al.*, 1988). Aliquots of membranes (50–100 μg of protein) were incubated for 1 h at 25°C with $[^{125}I]$EGF (final concentration 1 nM) in the presence and absence of unlabelled EGF (final concentration 100 nM) to correct for non-specific binding. Membranes were pelleted by centrifuging at 14,000 *g* for 7 min at 4°C, washed with ice-cold phosphate-buffered saline (PBS) containing 0.2% BSA, recentrifuged as above and counted in a Beckman Gamma 5500B spectrometer. Tumours with an EGFR level of over 20 fmol per mg of protein were considered positive.

*Statistical analysis*

To evaluate statistical significance the chi-square and Fisher exact tests were applied as appropriate. A *P*-value of $<0.05$ was considered significant.

### Results

*Tissue distribution of immunochemical staining*

Of the 111 specimens stained, 74 had normal epithelium adjacent to areas of tumour. Of these, 72 (97%) were positive for *bcl*-2 (Figure 1a). Also, in the seven specimens of benign disease stained, both affected and normal epithelium was also positive for *bcl*-2. However, in the areas of invasive tumour, only 88 of the 111 tumours expressed *bcl*-2 (Figure 1c), 79% of the total, leaving 23 tumours or 21% *bcl*-2 negative (Figure 1d). In addition, of the 88 positive tumours, 12 did display some areas of focal loss of expression. Lymphocytes, which always expressed *bcl*-2, and areas of adjacent normal tissue were utilised as in-built positive controls in cases where the tumour was found not to express *bcl*-2 (Figure 1b). Connective tissue fibroblasts did not express *bcl*-2 in any of the specimens studied.

Forty-three of the 111 invasive tumours also contained areas of *in situ* disease, which was assessed separately (Table I). Of 38 patients with *bcl*-2-positive invasive disease, 34 (90%) also had *bcl*-2-positive *in situ* areas. Of the five patients with *bcl*-2-negative invasive disease, all were likewise negative in their *in situ* areas ($P = 0.0001$).

*Node status*

In breast disease the single most powerful predictor of outcome is axillary node status. Of our 111 patients, 108 have had axillary node dissections and a comparison of the results is shown in Table II. Sixty-two of 108 (57%) were found to be node positive. Fifty-two of 85 (61%) *bcl*-2-positive patients were node positive. Of the 23 *bcl*-2-negative patients, ten (43%) were node positive (no significant difference: $P = 0.199$).

*Other clinical parameters*

Of the series of tumours stained for *bcl*-2, 107 had their size at excision recorded. The average size of the 85 *bcl*-2-positive tumours was 25 (± 1) mm compared with an average of 29 (± 2) mm in the 22 *bcl*-2-negative tumours. Similarly, no difference could be seen in *bcl*-2 expression in tumours of ⩽ 10 mm compared with those that were larger ($P = 0.682$). Nor could any correlation be found between *bcl*-2 expression and tumour type ($P = 0.315$ when comparing ductal carcinomas with all other types) or degree of differentiation ($P = 0.702$). The prevalence of *bcl*-2 expression in the tumours of pre- and post-menopausal women is roughly similar, 76% in women of 50 and younger and 81% in women over 50; no significant difference is evident ($P = 0.681$) (Table II).

*Receptor status*

All 111 patients in our series had accompanying ER and EGFR status recorded. Breast carcinomas were considered positive for ER if they contained > 5 fmol of ER per mg of protein. Comparing *bcl*-2 expression with ER status using this cut-off point, a close relationship of expression was observed.

Seventy of 88 *bcl*-2-positive tumours (80%) were also ER positive, whereas of the *bcl*-2-negative tumours only seven out of 23 (30%) were positive for ER ($P < 0.0001$) (Table III).

Tumours containing > 20 fmol of EGFR per mg of protein were considered positive for EGFR. Twenty-six out of 88 *bcl*-2-positive tumours were EGFR positive (30%), compared with 16 out of 23 *bcl*-2-negative tumours (70%). This was the converse of the findings for ER ($P = 0.001$) (Table III).

Using ER and EGFR status, it is possible to divide breast cancer patients into four distinct prognostic groups (Sainsbury *et al.*, 1987). Patients with $ER^+/EGFR^-$ tumours have the best prognosis. Prognosis is poorer for patients with the $ER^-/EGFR^-$ followed by patients with $ER^+/EGFR^+$

**Figure 1** **a**, *bcl*-2-positive benign epithelium of normal duct (n) (bar = 100 μm). **b**, *bcl*-2-positive benign epithelium (n) and positive lymphocytes (l) with negative tumour (t) (bar = 100 μm). **c**, *bcl*-2-positive tumour (t) surrounded by negative connective tissue (bar = 50 μm). **d**, *bcl*-2-negative tumour (t) with positively stained lymphocytes (l) (bar = 50 μm).

tumours; finally, $ER^-/EGFR^+$ tumours are associated with the worst prognosis. Comparing these groups (shown in Table IV), both ER-positive groups have the highest proportion of *bcl*-2-positive tumours (93% and 86% respectively). However, the most marked down-regulation of *bcl*-2 expression is in ER-negative tumours which are also EGFR positive (38%) ($P < 0.0001$ when compared with other three groups combined), suggesting an inhibitory role for EGFR on *bcl*-2 expression.

### c-erb*B-2 and p53*

c-*erb*B-2 protein is another receptor in the EGFR family. Expression of c-*erb*B-2 was rare in *bcl*-2-positive tumours

**Table I** *bcl*-2 expression in *in situ* areas of invasive breast carcinoma ($P = 0.0001$)

| | bcl-*2-positive* in situ *disease* | bcl-*2-negative* in situ *disease* |
|---|---|---|
| *bcl*-2-positive invasive disease | 34 | 4 |
| *bcl*-2-negative invasive disease | 0 | 5 |

**Table III** Expression of oestrogen receptor, epidermal growth factor receptor, *cerb*B-2 and p53 in *bcl*-2-positive and -negative primary invasive breast carcinomas

| | bcl-*2-positive invasive tumours* | bcl-*2-negative invasive tumours* | P *value* |
|---|---|---|---|
| ER positive | 70 | 7 | |
| ER negative | 18 | 16 | |
| ER positive (%) | 80 | 30 | <0.0001 |
| EGFR positive | 26 | 16 | |
| EGFR negative | 62 | 7 | |
| EGFR positive (%) | 30 | 70 | 0.001 |
| c-*erb*-B-2 positive | 2 | 6 | |
| c-*erb*-B-2 negative | 49 | 13 | |
| c-*erb*-B-2 positive (%) | 4 | 32 | 0.004 |
| p53 positive | 27 | 15 | |
| p53 negative | 52 | 6 | |
| p53 positive (%) | 34 | 71 | 0.005 |

**Table II** Comparison of *bcl*-2 expression in invasive breast carcinoma with node status ($P = 0.199$), tumour size at excision ($P = 0.682$), tumour type ($P = 0.315$), degree of differentiation ($P = 0.702$) and age at excision ($P = 0.681$)

| | *Node status* | | *Size* | | *Tumour type* | | | | *Differentiation* | | | *Age (years)* | |
|---|---|---|---|---|---|---|---|---|---|---|---|---|---|
| | *Positive* | *Negative* | *≤10 mm* | *>10 mm* | *Ductal* | *Lobular* | *Mixed* | *Other* | *Good* | *Mod-erate* | *Poor* | *≤50* | *>50* |
| *bcl*-2-positive invasive disease | 52 | 33 | 8 | 77 | 61 | 14 | 9 | 4 | 8 | 30 | 23 | 28 | 56 |
| *bcl*-2-negative invasive disease | 10 | 13 | 1 | 21 | 19 | 1 | 0 | 3 | 1 | 11 | 10 | 9 | 13 |

Table IV *bcl*-2 expression in receptor-defined prognostic groups

| | $ER^+/EGFR^-$ | $ER^-/EGFR^-$ | $ER^+/EGFR^+$ | $ER^-/EGFR^+$ |
|---|---|---|---|---|
| *bcl*-2-positive invasive tumours | 52 | 10 | 18 | 8 |
| *bcl*-2-negative invasive tumours | 4 | 3 | 3 | 13 |
| *bcl*-2 positive (%) | 93 | 77 | 86 | 38 |

(only 4%, Table III). However, expression was markedly higher in the *bcl*-2-negative tumours (32%) ($P = 0.004$).

When *bcl*-2 and p53 expression were compared, it was apparent that an inverse relationship existed between the two oncoproteins (Table III). One hundred tumours were stained for both and it was found that only 27 out of 79 *bcl*-2-expressing tumours also expressed p53 (34%); however, of the 21 *bcl*-2-negative tumours, 15 were p53 positive (71%) ($P = 0.005$).

### Discussion

*bcl*-2 has been shown to confer resistance to apoptotic cell death (Vaux *et al.*, 1988). It is apparent that in normal breast epithelium *bcl*-2 is nearly always expressed; this is also true of benign breast disease. In the normal breast, a continuous renewal system is in operation, which is partly controlled hormonally. This process progresses at a much slower rate than, for example, that seen in the gut. Other glandular organs with slow cell turnover rates also show *bcl*-2 expression, for example the thyroid (Hockenbery *et al.*, 1991). Occasionally epithelial cells that had been shed into the luminal space were negative for *bcl*-2. They had probably lost *bcl*-2 expression and were now in the process of undergoing apoptosis.

A tumour that is derived from breast epithelial cells might also be expected to express *bcl*-2. Apoptosis is a feature commonly seen in tumours, and in fact the ability to resist apoptosis may seem to offer an advantage to a rapidly growing tumour, by slowing down the cell loss rate.

We have observed that *bcl*-2 is expressed in 79% of invasive breast carcinomas, and that this expression is generally mirrored in adjacent *in situ* disease when it is present. Since 97% of normal breast epithelium specimens, 91% of *in situ* cancer specimens and 79% of tumours express *bcl*-2, it can be concluded that loss of *bcl*-2 expression, when it occurs, is a relatively late event in the progression of the disease. This is the converse of the observation of *erb*B-2, which is present *in situ* and lost in invasive cancer (Barnes *et al.*, 1988). However, in a few cases, there is loss of *bcl*-2 from *in situ* cancer but not from adjacent invasive cancer. The explanation may be that in these cases the invasive element has arisen from a different *in situ* component to that studied in adjacent areas.

It has been suggested that p53 and *bcl*-2 have opposite functions: that p53 is a death pathway gene (Yonisch-Rouach *et al.*, 1991; Shaw *et al.*, 1992) and that *bcl*-2 is an antidote to programmed cell death (Hockenbery *et al.*, 1990). Aberrations in either function could lead to extended survival of neoplastic cells and the increased likelihood of mutational aberrations in other oncogenes, such as those responsible for growth and proliferation or tumour-suppressor genes. We found that there is an inverse relationship between *bcl*-2 and p53. It is possible that either one or the other is sufficient to modify the apoptosis pathway in solid tumours.

It has been noticed previously that *bcl*-2 expression is associated with glandular epithelium in which regulation of hyperplasia and involution is achieved hormonally or by growth factors. The breast is a prime example of such tissue, with both oestrogen and epidermal growth factor playing important roles in the regulation of breast epithelium. Indeed, the levels of oestrogen receptor (ER) and epidermal growth factor receptor (EGFR) are in themselves predictors of prognosis, ER being associated with good prognosis (Howell *et al.*, 1984) and EGFR associated with poor prognosis (Sainsbury *et al.*, 1987). Eighty per cent of *bcl*-2-positive tumours were also ER positive, compared with only 30% in the *bcl*-2-negative group. This raises the possibility that *bcl*-2 could be an oestrogen-regulated protein. A similar finding has been reported by Chan *et al.* (1993) in a series of 41 patients with breast carcinoma. Consistently, a negative relationship was found between *bcl*-2 and EGFR and c-*erb*B-2. Although *bcl*-2 expression was correlated with ER, it was in the $EGFR^+/ER^-$ group, rather than the $EGFR^-/ER^-$ group, that the inverse relationship was most marked. This suggests that EGFR ligands may have a role in down-regulation of *bcl*-2.

In conclusion, the normal biological mechanism of action of *bcl*-2 is as yet unknown. It does appear, however, that it may be under hormonal control, acting as a regulator of cellular events until it is switched off. At this point in normal tissue, apoptosis would occur. In neoplastic tissues the reduction in expression of *bcl*-2 may require alternative survival pathways. If such cells are thereby relatively more resistant to apoptosis, this may contribute to their resistance to therapy and environmental stress, hence a poorer prognosis. Loss of *bcl*-2 expression was associated with a range of other poor prognostic markers including EGFR, c-*erb*B-2 and p53 positivity. Thus its role in breast cancer progression may differ substantially from that seen in lymphoma, in which activation by translocation occurs. *bcl*-2 is universally expressed in normal breast epithelium, and a subset of tumours lose expression at a later stage in their progression; this group is associated with the established molecular markers of poor prognosis. However, whether *bcl*-2 expression will prove to be an independent marker of prognosis in neoplastic breast disease remains to be seen.

### References

BAKHSHI, A., JENSEN, J.P., GOLDMAN, P., WRIGHT, J.J., MCBRIDE, O.W., EPSTEIN, A.L. & KORSMEYER, S.J. (1985). Cloning the chromosomal breakpoint of t(14;18) human lymphomas; clustering around $J_H$ on chromosome 14 and near a transcriptional unit on 18. *Cell*, **41**, 899-906.

BARNES, D.M., LAMMIE, G.A., MILLIS, R.R., GULLICK, W.L., ALLEN, D.S. & ALTMAN, D.G. (1988). An immunohistochemical evaluation of c-*erb*B-2 expression in human breast carcinoma. *Br. J. Cancer*, **58**, 448-452.

BRADFORD, M.M. (1976). A rapid and sensitive method for the quantification of microgram quantities of protein utilizing the principle of protein dye binding. *Anal. Biochem.*, **72**, 248-254.

CHAN, W.K., POULSOM, R., LU, Q.L., PATEL, K., GREGORY, W., FISHER, C.J. & HANBY, A.M. (1993). Bcl-2 expression in invasive mammary carcinoma: correlation with apoptosis, hormone receptors, and p53 expression. *J. Pathol.*, **169** (Suppl.), 138.

CHEN-LEAVY, Z., NOURSE, J. & CLEARY, M. (1989). The bcl-2 candidate proto-oncogene product is a 24-Kilodalton integral-membrane protein highly expressed in lymphoid cell lines and lymphomas carrying the t(14;18) translocation. *Mol. Cell. Biol.*, **9**, 701-710.

CLEARY, M.L. & SKLAR, J. (1985). Nucleotide sequence of a t(14;18) chromosomal breakpoint in follicular lymphoma and demonstration of a breakpoint cluster region near a transcriptionally active locus on chromosome 18. *Proc. Natl. Acad. Sci. USA*, **82,** 7439-7443.

CLEARY, M.L., SMITH, S.D. & SKLAR, J. (1986). Cloning and structural analysis of cDNAs for bcl-2 and a hybrid bcl-2/immunoglobulin transcript resulting from the t(14;18) translocation. *Cell*, **47,** 19-28.

CORBETT, I.P., HENRY, J.A., ANGUS, B., WATCHORN, C.J., WILKINSON, L., HENNESSY, C., GULLICK, W.J., TUZI, N.L., MAY, F.E.B., WESTLEY, B.R. & HORNE, C.H.W. (1990). NCL-CB11, a new monoclonal antibody recognizing the internal domain of the c-erbB-2 oncogene protein effective for use on formalin-fixed, paraffin-embedded tissue. *J. Pathol.*, **161,** 15-25.

CORDELL, J.L., FALINI, B., ERBER, W.N., GHOSH, A.K., ABDULAZIZ, Z., MACDONALD, S., PULFORD, K.A.F., STEIN, H. & MASON, D. (1984). Immunoenzymatic labelling of monoclonal antibodies using immune complexes of alkaline phosphatase and monoclonal anti-alkaline phosphatase (APAAP complexes). *J. Histochem. Cytochem.*, **32,** 219-229.

EORTC BREAST CANCER CO-OPERATIVE GROUP (1980). Revision of the standards for the assessment of hormone receptors in human breast cancer. *Eur. J. Cancer*, **16,** 1513-1515.

GANNON, J.V., GREAVES, R., IGGO, R. & LANE, D.P. (1990). Activating mutations in p53 produce common conformational effects. A monoclonal antibody specific for the mutant form. *EMBO J.*, **9,** 3927-3934.

GRANINGER, W.B., SETO, M., BOUTAIN, B., GOLDMAN, P. & KORSMEYER, S.J. (1987). Expression of bcl-2 and bcl-2 fusion transcripts in normal and neoplastic cells. *J. Clin. Invest.*, **80,** 1512-1515.

HARRIS, A.L. (1992). p53 expression in human breast cancer. *Adv. Cancer Res.*, **59,** 69-87.

HOCKENBERY, D., NUNEZ, G., MILLIMAN, C., SCHREIBER, R.D. & KORSMEYER, S.J. (1990). Bcl-2 is an inner mitochondrial membrane protein that blocks programmed cell death. *Nature*, **348,** 334-336.

HOCKENBERY, D.M., ZUTTER, M., HICKEY, W., NAHM, M. & KORSMEYER, S.J. (1991). Bcl-2 protein is topographically restricted in tissues characaterized by apoptotic cell death. *Proc. Natl Acad. Sci. USA*, **88,** 6961-6965.

HOWELL, A., BARNES, D.M., HARLAND, R.N.L., REDFORD, J., BRAMWELL, V.H.C., WILKINSON, M.J.S., SWINDELL, R., CROWTHER, D. & SELLWOOD, R.A. (1984). Steroid-hormone receptors and survival after first relapse in breast cancer. *Lancet*, **1,** 588-591.

JACOBSON, M.D., BURNE, J.F., KING, M.P., MIYASHITA, T., REED, J.C. & RAFF, M.C. (1993). Bcl-2 blocks apoptosis in cells lacking mitochondrial DNA. *Nature*, **361,** 365-369.

MCGUIRE, W.L. & CLARK, G.M. (1992). Prognostic factors and treatment decisions in axillary-node-negative breast cancer. *N. Engl. J. Med.*, **326,** 1756-1761.

NICHOLSON, S., SAINSBURY, J.R.C., NEEDHAM, G.K., CHAMBERS, P., FARNDON, J.R. & HARRIS, A.L. (1988). Quantitive assays of epidermal growth factor receptor in human breast cancer: cut off points of clinical relevance. *Int. J. Cancer*, **42,** 36.

NUNEZ, G., LONDON, L., HOCKENBERY, D., ALEXANDER, M., MCKEARN, J.P. & KORSMEYER, S.J. (1990). Deregulated bcl-2 gene expression selectively prolongs survival of growth factor-deprived hemopoietic cell lines. *J. Immunol.*, **144,** 3602-3610.

PEZZELLA, F., MORRISON, H., JONES, M., GATTER, K.C., LANE, D., HARRIS, A.L. & MASON, D.Y. (1992*a*). Immunohistochemical detection of p53 and bcl-2 proteins in non-Hodgkins lymphoma. *Histopathology*, **22,** 39-44.

PEZZELLA, F., JONES, M., RALFKIER, E., ERSBØLL, J., GATTER, K.C. & MASON, D.Y. (1992*b*). Evaluation of bcl-2 protein expression and 14;18 translocation as prognostic markers in follicular lymphoma. *Br. J. Cancer*, **65,** 87-89.

SAINSBURY, J.R.C., FARNDON, J.R., NEEDHAM, G.K., MALCOLM, A.J. & HARRIS, A.L. (1987). Epidermal growth factor status as predictor of early recurrence of and death from breast cancer. *Lancet*, **1,** 1398-1402.

SHAW, P., BOVEY, R., TARDY, S., SAHLI, R., SORDAT, B. & COSTA, J. (1992). Induction of apoptosis by wild-type p53 in a human colon tumor-derived cell line. *Proc. Natl Acad. Sci. USA*, **89,** 4495-4499.

TSUJIMOTO, Y. & CROCE, C.M. (1986). Analysis of the structure, transcripts and protein products of bcl-2, the gene involved in human follicular lymphoma. *Proc. Natl Acad. Sci. USA*, **83,** 5214-5218.

TSUJIMOTO, Y., GORHAM, J., COSSMAN, J., JAFFE, E. & CROCE, C.M. (1985). The t(14;18) chromosome translocations involved in B-cell neoplasms result from mistakes in VDJ joining. *Science*, **299,** 1390-1393.

TSUJIMOTO, Y., IKEGAKI, N. & CROCE, C.M. (1987). Characterization of the protein product of bcl-2, the gene involved in human follicular lymphoma. *Oncogene*, **2,** 3-7.

VAUX, D.L., CORY, S. & ADAMS, J.M. (1988). Bcl-2 gene promotes haemopoietic cell survival and cooperates with c-myc to immortalize pre-B cells. *Nature*, **335,** 440-442.

VOGELSTEIN, B. & KINZLER, W. (1992). p53 Function and dysfunction. *Cell*, **70,** 523-526.

WRIGHT, C., ANGUS, B., NICHOLSON, S., SAINSBURY, J.R.C., CAIRNS, J., GULLICK, W.J., KELLY, P., HARRIS, A.L. & HORNE, C.H.W. (1989). Expression of c-erb B-2 oncoprotein: a prognostic indicator in human breast cancer. *Cancer Res.*, **49,** 2087-2090.

YONISH-ROUACH, E., RESNITZKY, D., LOTEM, J., SACHS, L., KIMCHI, A. & OREN, M. (1991). Wild-type p53 induces apoptosis of myeloid leukaemic cells that is inhibited by interleukin-6. *Nature*, **352,** 345-347.